# Population and conservation genomics of forest trees: seeing the forest for the trees

**DOI:** 10.1186/1753-6561-5-S7-I2

**Published:** 2011-09-13

**Authors:** Andrew Eckert

**Affiliations:** 1 Department of Ecology and Evolution, University of California at Davis, Davis, CA, 95616, USA

## Background

Forest trees exhibit striking adaptations to the environments in which they grow. A long history of quantitative genetic experimentation has established the genetic basis for many traits, which are likely adaptive since many of them are also correlated with environmental heterogeneity. The genes underlying these traits, however, have largely remained elusive. Recent applications of high-throughput sequencing and genotyping technologies to natural populations of forest trees have identified several promising candidates for genes underlying complex and adaptive traits (reviewed by [1]). The diversity of analytical approaches employed in those studies, however, begs the question of the generality of reported results. Here, I exploited the diversity of analytical approaches used previously to identify genes underlying adaptive traits for two conifer species to assess the logical consistency among results generated from different conceptual frameworks.

## Materials and methods

**Figure 1:**
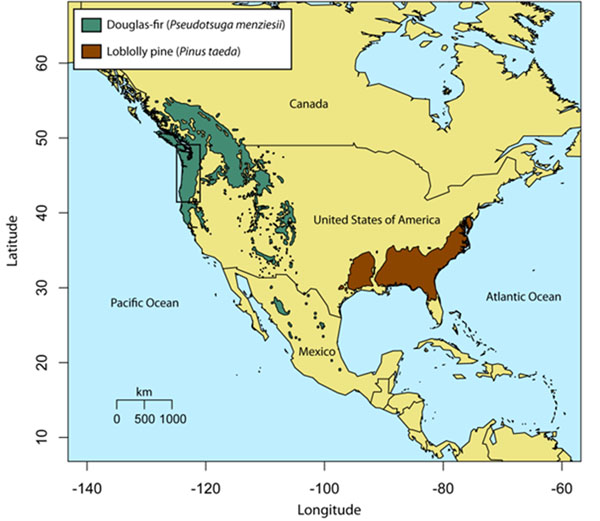
The distribution of Douglas-fir and loblolly pine in North America. The black box surrounds the sampling area for Douglas-fir, while the entire range for loblolly pine was sampled.

Data sets comprised of genotyped single nucleotide polymorphisms (SNPs) were gathered for two North American conifers (Fig. 1): loblolly pine (*Pinus taeda* L.) and coastal Douglas-fir (*Pseudotsuga menziesii* (Mirb.) Franco var. *menziesii*). These data sets were generated through resequencing of diversity panels (n = 18-24 megagametophytes) for a sample of expressed sequence tag (EST) unigenes (*n* = 7,535) and cold-hardiness related candidate genes (*n* = 121), respectively. Genotyping was performed using Illumina’s Infinium (loblolly pine) or GoldenGate (coastal Douglas-fir) array technologies. For each data set, associations to phenotypes and environmental variables were gathered from the literature or performed as described elsewhere (see references in [1]). For each associated gene, I asked two questions: (1) Are genes associated to phenotypes more often also associated to environmental variables than randomly chosen genes? (2) Are associated genes also outliers for nucleotide diversity and site-frequency spectrum based statistics, nucleotide divergence or *F*_ST_ more often than randomly chosen genes? Permutation tests were used to assess whether or not observed patterns were different than those produced by chance.

## Results

**Figure 2:**
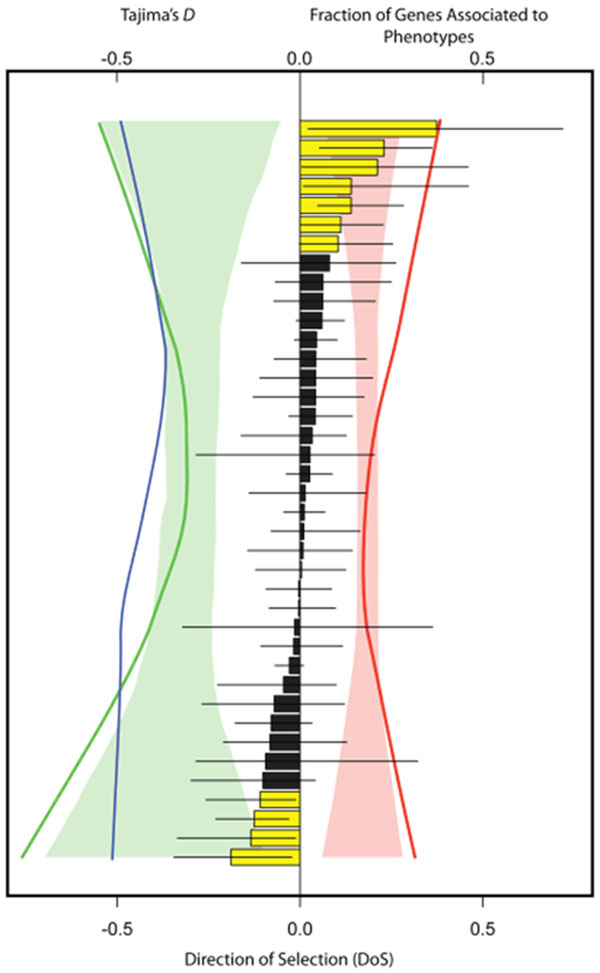
An example of logical consistency for loblolly pine between genes associated to phenotypes or environmental variables and gene categories that have average deviations from neutrality consistent with positive or negative selection. Error bars give 99% bootstrap confidence intervals for the direction of selection statistic (DoS), with yellow bars having confidence intervals excluding zero. Lines give average Tajima’s *D* for nonsynonymous (blue) and synonymous sites (green), as well as the proportion of genes associated to at least one phenotype (red). Shaded areas give null distributions (99% quantiles) generated via permutations of genes among categories (*n* = 10,000 permutations). All lines, including those forming the null distributions, were smoothed using lowess smoothing.

A total of ~30 genes were associated with cold-hardiness phenotypes or with climate data for coastal Douglas-fir, while ~850 associated genes were identified for loblolly pine. Genes associated with phenotypes for coastal Douglas-fir were more often associated to environmental variables than randomly chosen genes (*P* = 0.005), which was only moderately apparent for loblolly pine (*P* = 0.067). Genes associated to phenotypes or environmental variables were not more likely to be outliers for nucleotide diversity, site-frequency spectrum based statistics, nucleotide divergence or *F*_ST_ for Douglas-fir (*P* > 0.05). There was some evidence, however, for non-neutral evolution for associated genes using a statistic based on the McDonald-Kreitman table [2]. In this case, associated genes had too many extreme values for the direction of selection (DoS) statistic than expected from randomly resampling the available genes (*P* < 0.05). Associated genes on average had skewed site-frequency spectra for loblolly pine, especially for synonymous sites, as well as too many extreme values for the DoS statistic. Further classification of genes for loblolly pine into functional categories revealed striking trends indicative of non-neutral processes underlying some of the associations (Fig. 2). A total of 11 gene categories were consistent with positive (*n* = 7) or negative (*n* = 4) selection. In both cases, the frequency of associated loci increased for these categories and the synonymous site frequency spectra became more skewed. Many associations for loblolly pine may thus reflect linked selection, with molecular phenotypes (e.g. gene expression) accounting for all the associations in gene categories indicative of negative selection and environmental associations (e.g. aridity) being largely located in gene categories consistent with positive selection.

## Conclusions

Many of the genes associated to phenotypes for both species were also correlated to environmental variables and exhibited patterns of non-neutral evolution. Thus, associated genes are prime targets for conservation efforts. The questions posed here, however, make the strong assumption that genes associated to phenotypes or environmental variables should also show non-neutral patterns of evolution. This is not always expected to be the case [3,4], yet the lack of consistency is often interpreted as such and is one explanation for those genes or sets of genes reported here as lacking non-neutral signals. The search for logical consistency among analytical approaches, however, often focuses on uninformative patterns. To illustrate this point, I employed a novel environmental association approach that correlates genetic divergence to environmental change and show that most of the site-frequency spectrum based outliers for coastal Douglas-fir [5] are correlated to change in climate variables but not to extant climate patterns. Taken together these results illustrate that non-neutral genes are often identified during association analyses, that departures from neutrality for genes driving associations are not only those due to recent directional selection, and that further work is needed to understand the population genetic processes underlying associations between genotypes, phenotypes and the environment.
